# Investigating the effects of context, visual working memory, and inhibitory control in hybrid visual search

**DOI:** 10.3389/fnhum.2024.1436564

**Published:** 2024-08-27

**Authors:** Alessandra Barbosa, Gonzalo Ruarte, Anthony J. Ries, Juan E. Kamienkowski, Matias J. Ison

**Affiliations:** ^1^School of Psychology, University of Nottingham, Nottingham, United Kingdom; ^2^Laboratorio de Inteligencia Artificial Aplicada, Instituto de Ciencias de la Computación (Universidad de Buenos Aires – Consejo Nacional de Investigaciones Científicas y Técnicas), Buenos Aires, Argentina; ^3^DEVCOM Army Research Laboratory, Aberdeen Proving Ground, MD, United States; ^4^Departamento de Computación (Facultad de Ciencias Exactas y Naturales, Universidad de Buenos Aires), Buenos Aires, Argentina

**Keywords:** visual search, memory search, real-world scenes, individual differences, visual working memory capacity, inhibitory control

## Abstract

**Introduction:**

In real-life scenarios, individuals frequently engage in tasks that involve searching for one of the distinct items stored in memory. This combined process of visual search and memory search is known as hybrid search. To date, most hybrid search studies have been restricted to average observers looking for previously well-memorized targets in blank backgrounds.

**Methods:**

We investigated the effects of context and the role of memory in hybrid search by modifying the task’s memorization phase to occur in all-new single trials. In addition, we aimed to assess how individual differences in visual working memory capacity and inhibitory control influence performance during hybrid search. In an online experiment, 110 participants searched for potential targets in images with and without context. A change detection and go/no-go task were also performed to measure working memory capacity and inhibitory control, respectively.

**Results:**

We show that, in target present trials, the main hallmarks of hybrid search remain present, with a linear relationship between reaction time and visual set size and a logarithmic relationship between reaction time and memory set size. These behavioral results can be reproduced by using a simple drift-diffusion model. Finally, working memory capacity did not predict most search performance measures. Inhibitory control, when relationships were significant, could account for only a small portion of the variability in the data.

**Discussion:**

This study provides insights into the effects of context and individual differences on search efficiency and termination.

## 1 Introduction

Visual search (*VS*) is the action of looking for a target among distractors. It is a ubiquitous task in many everyday situations, from searching for products in stores to driving. Mainly used to examine visual attention (Treisman and Gelade, 1980), visual search has been intensively researched for decades. The core manipulation in visual search is to vary the distractor set size while measuring the time until a target is detected (reaction times or RTs) and measuring detection accuracy (Neisser, 1964). From these, a hallmark was established—the linear dependence between RT and visual set size (VSS) (Wolfe, 2020).

While much is known about elements that influence visual search when *one* sole item is searched for, search in real life is substantially more complex, often involving several objects in memory. From (Schneider and Shiffrin, 1977) classic work, encompassing both visual search and memory search (MS), hybrid search (HS) is when observers search for any of many possible targets (Wolfe, 2012a). Hybrid search tasks involve memorizing potential targets to subsequently identify their incidence in a display. In a previous study (Schneider and Shiffrin, 1977), different memory manipulations included whether the hybrid search had consistent mapping (when target sets are fixed, used throughout trials), all-new mapping (when new items compose the memory set each trial), or varied mapping (when targets become distractors and vice versa), which negatively impacted efficiency and accuracy in this order. Regardless of the specific conditions, a robust logarithmic relationship between RT and memory set size (MSS) has been consistently reported: up to MSSs of 100 for objects (Wolfe, 2012a) and words (Boettcher and Wolfe, 2015). Nonetheless, most recent hybrid search studies have not manipulated memory mappings. Solely utilizing consistent-mapping-like paradigms, high accuracies are observed (e.g., see Wolfe, 2012a). Under such conditions, speed-accuracy trade-offs that vary across set sizes cannot be evaluated as adequately as by paradigms that limit memory strengths (Nosofsky et al., 2014b). Given the prominent role of context in memory representations, such as recognizing a face or place after a single real-world encounter (Ison et al., 2015), examining paradigms with trial-by-trial changing memory sets is justified.

Most hybrid search findings are based on experiments conducted with artificial stimuli on blank backgrounds (Wolfe et al., 2011b), which may limit their ecological validity. In visual search, accumulating evidence shows that context is critical in guiding attention in real-world search (Wolfe et al., 2011a). Indeed, context guidance was found to overpower bottom-up saliency in guiding eye movements in naturalistic search (Henderson et al., 2009) and facilitate search in scenes for targets in plausible locations rather than implausible ones (Neider and Zelinsky, 2006) or blank backgrounds (Wolfe et al., 2011a). However, naturalistic scenes are complex/continuous, with set sizes impossible to define (Rosenholtz et al., 2007) but they are never random (Henderson and Ferreira, 2004). Scenes have syntax, that is, structural plausibility—humans appear on horizontal superficies (Biederman, 1977; Torralba et al., 2006), and semantics, that is, meaningful associations—for example, a toothbrush on a sink (Võ and Henderson, 2009). In hybrid search, the limited exploration of contextual information has left mixed results. To our knowledge, only Boettcher et al. (2013, 2018) attempted to examine contextual effects in hybrid search. However, both were investigating whether context-aided memory set partition to context-relevant items at *fixed visual set size* and memory set size. This means that it is largely unknown how variable set sizes affect their relationship with context. The primary objective of this study was to explore the relationship between reaction time and set size in visual search and memory search.

A small number of hybrid search models have been proposed in the literature to explain behavioral signatures in hybrid search. Cunningham and Wolfe (2014) postulated a three-stage model for hybrid search. After the first stage of *guided* search (Wolfe, 2007), where solely feature-plausible objects affiliated to the memory set are considered for the second stage of object recognition, a third stage of logarithmic memory search is performed through a diffusion process. Each memorized target item races as information is accumulated in parallel over time, forming N-diffusion processes, where *N* = MSS. Target selection only occurs when information from a target accumulator reaches the decision threshold. The threshold is set higher to impede exaggerated false alarms and lower to promote speed—the speed-accuracy trade-off (Chun and Wolfe, 1996). As most hybrid search paradigms follow a condition akin to consistent mapping, not much is known when targets’ memory strengths are manipulated (Wolfe et al., 2015). Drawing from memory search literature, which focuses on the processes underlying memory search through memory-mapping manipulations, Nosofsky et al. (2014b) proposed an exemplar-familiarity random-walk memory search model with a logarithmic RT diffusion stage. In contrast to recent hybrid search models, it proposes that racing items are dictated by their ‘memory strength’, influenced by repetition effects and time presented, while they compete, and information is accumulated. Thus, while the curvilinear relationship of RTxMSS is maintained, efficiency and accuracy are affected (Nosofsky et al., 2014a,b), which can elucidate hybrid search processes for different memorization conditions. Another family of computational models that have been extensively used in decision-making tasks involving two-choice decision-making is the drift-diffusion model (DDM) (Ratcliff and McKoon, 2008). Essentially, the decision-maker accumulates evidence until a boundary is reached, and at that moment the decision is made. This process is modeled by sequential diffusion signals that drift over time. Diffusion models have been used to estimate the RT distribution in visual search tasks before (Palmer, 1975), and they may provide valuable insights into the underlying cognitive processes (Myers et al., 2022). More complex models have emerged in recent years that try to predict human scanpaths in a visual search task and one could also sample RTs from them (Kummerer et al., 2018; Bujia et al., 2022; Travi et al., 2022). The problem with this approach is that the more complex the model is, the more difficult it is to understand its underlying processes and relate them to a particular study. A secondary objective in this study was to implement a drift-diffusion model and interpret its parameter values (drift, boundary, non-decision time) in terms of the hybrid search variables, such as the visual set size (VSS), the memory set size (MSS), and the presence of the context.

Two fundamental components of executive function, namely, working memory and inhibitory control, have long been largely implicated in visual search processes. Visual working memory (VWM), which entails the maintenance and manipulation of a limited amount of visual information that serves current task demands (Luck and Vogel, 1997), has been connected to a variety of visual search processes, such as representing the search template to guide attention (Desimone and Duncan, 1995), comparing the search template to potential suitor objects (Bundesen, 1990), and influencing search facilitation in visual search under contextual cueing (Manginelli et al., 2013). Most recent hybrid search research has emulated a consistent mapping paradigm, which has been seen to produce near-error-free data (Nosofsky et al., 2014b). Studying different memory manipulations could be critical to further uncover memory’s role in search, since visual working memory involvement might only be observed when targets change per trial (Woodman et al., 2007), and in memory search it is still unclear if context can restrict the memory set to scene-relevant items on a trial-by-trial basis (Boettcher et al., 2013, 2018). Inhibitory control (IC) is the critical executive function of suppressing goal-irrelevant stimuli interference and subduing prepotent motor response (Young et al., 2018). Experimental paradigms conventionally include brusque prepotent response incitation, where one either proceeds or subdues action (e.g., go-no-go tasks) (Miyake et al., 2000). Individuals who have higher false alarms, or larger negative response bias, when subjected to signal detection theory (SDT) analysis, have more difficulty inhibiting prepotent responses (Young et al., 2018). Given the proposed importance of inhibitory control in visual search models for top-down selection and search termination (Treisman and Sato, 1990; Moran et al., 2013), an evaluation of the potential impact of individuals’ inhibitory control is also merited. The last objective of this study was to examine the role of individual differences in working memory and inhibitory control in hybrid search. Based on previous literature, it is feasible to formulate specific predictions regarding the manipulations employed in this study. With mounting replications of hybrid search’ RT signatures in divergent conditions, we expect all-new memorization and context to preserve *qualitatively* the main behavioral hybrid search signatures. However, we expect to see changes in search efficiency and accuracy. Predicting the role of visual working memory in hybrid search is more difficult, given the conflicting findings reported in visual search. If this study follows (Drew et al., 2016) account that a fixed amount of visual working memory is used as a conduit to transfer incoming target templates to memory, one might expect working memory capacity to correlate with RT intercepts in hybrid search, where individuals with higher working memory capacity might transfer targets through working memory faster than lower visual working memory capacity individuals. However, given our modified paradigm with targets changing per trial, analogous to Woodman et al. (2007), this proposition might not hold, and we might see higher visual working memory capacity producing smaller RT slopes as well; given high-visual working memory capacity individuals would have higher storage capacity/resource allocation flexibility as set sizes increase (Luck and Vogel, 2013). Given the potential importance of inhibitory control in search termination (Moran et al., 2013), we would expect that individuals with higher inhibitory control show larger RT intercepts than lower inhibitory control individuals, reflecting a potential to better maintain conservative thresholds with fewer false alarms and higher accuracy. In addition, by implementing a drift-diffusion model for hybrid search, we expect to link model parameters with the behavioral results and, in doing so, propose new directions for future experiments.

The rest of the study is organized as follows. Section 2 presents the results of the main experiment (Experiment 1) on the role of context in hybrid search. Section 3 shows that a simple drift-diffusion computational model can reproduce the main behavioral signatures of Experiment 1. Section 4 evaluates the extent to which individual differences in visual working memory and inhibitory control contribute to hybrid search behavior.

## 2 Experiment 1: context effects on hybrid search

### 2.1 Methods

#### 2.1.1 Participants

An online data collection method gathered data from 110 participants, identifying as women (59), men (49), non-binary (1), different identity (0), and non-disclosing (1). Participants were recruited via email and social media. Their ages (excluding 3 age misreports) ranged from 18 to 61 years old (*M =* 27 and *SD =* 7.63). Data were also collected for another 10 participants who were excluded due to aborting the experiment (*N* = 7) or low behavioral performance (*N* = 3). More specifically, to exclude participants with poor performance we used a threshold of 3 standard deviations below the mean accuracy (calculated from the raw data in the whole experiment), as done in previous visual search studies (Treisman, 1982). Participants were given the option to enter a draw for Amazon vouchers worth £20 each, so two people were compensated. Convenience sampling was adopted, as email and social media outreach is limited to users and not randomized. Online recruitment concentrated on Chinese-oriented social media platforms (WeChat) and Brazilian social media groups. The study was approved by the University of Nottingham School of Psychology Ethics Panel (ethics approval: S1240).

#### 2.1.2 Materials

**Figure 1:**
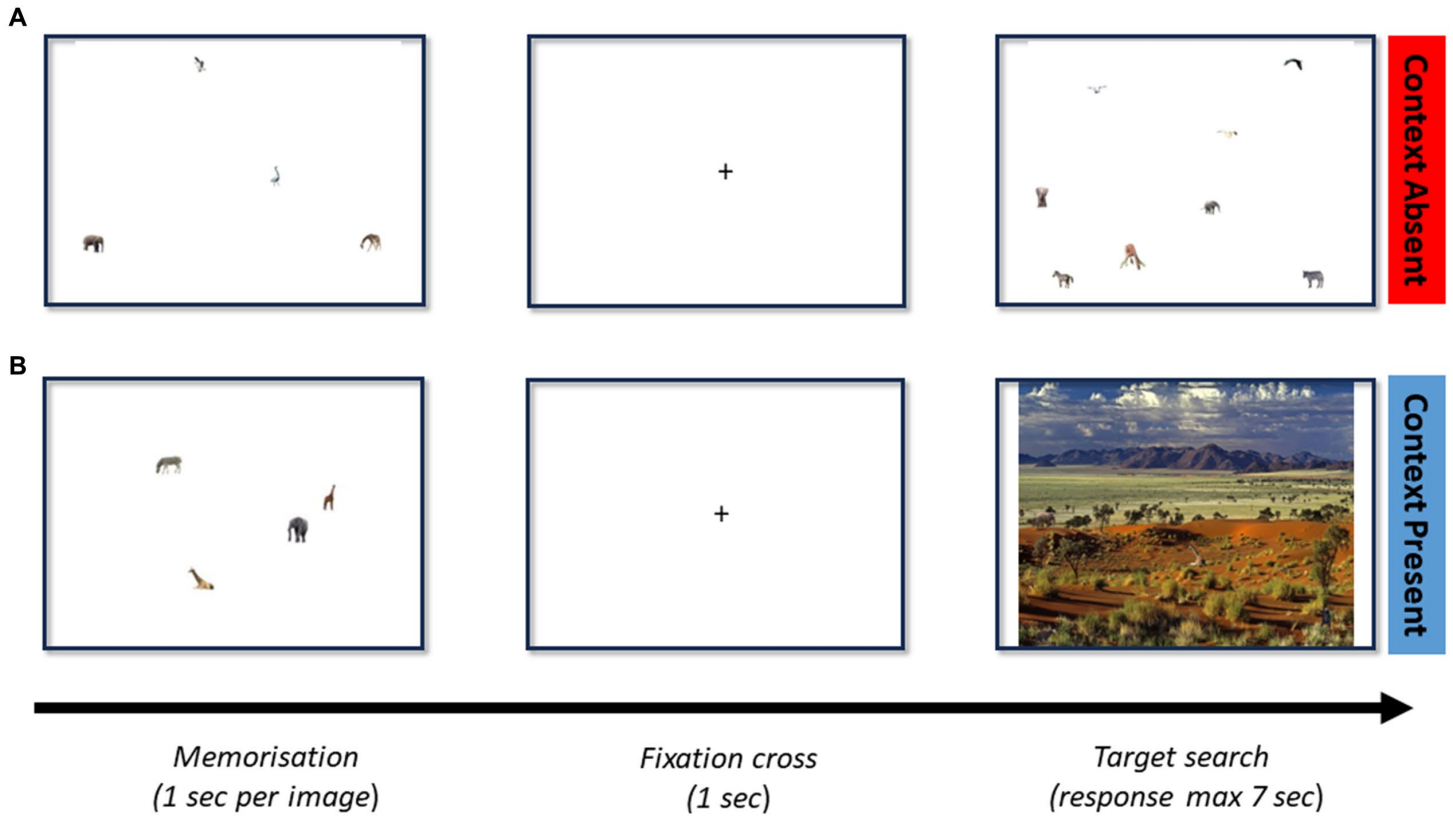
**(A)** Model trial for a memory set size of 4 and a visual set size of 8 without contextual information. **(B)** Model trial for a memory set size of 4 and a visual set size of 4 with context. Each trial initiates with the images to be memorized, which is then followed by a fixation cross. The search screen (containing or not the target) follows until a response is made.

A total of 112 images with (*N* = 56) and without (*N* = 56) context were constructed. As shown in Figure 1B, the contextual images were constructed by superimposing real-world background scenes (including outdoor scenes—e.g., forest, and indoor scenes—e.g., shelf) with target and distractor stimuli. Each image contained 1, 2, 4, or 8 stimuli (visual set size, VSS), and a separate set of 1, 2, 4, or 8 stimuli were selected for memorization (memory set size, MSS). Items (targets and distractors) only appeared once during the experiment. All the targets and distractor stimuli were built from images from the COCO dataset (Lin et al., 2014) and ImageNet (Deng et al., 2009). These datasets were selected due to their extensive size and appropriate licensing. Images were then resized so that their new size would be compatible with the background image and placed according to scene syntax (i.e., no major violations of support, interposition, position, and size). All targets and distractors belonged to the same category (objects, animals, or people). The experiments were implemented in PsychoPy and executed online by participants through https://pavlovia.org/ (Peirce et al., 2019).

#### 2.1.3 Procedure

Participants searched 112 images with or without context and varying MSS and VSS. Following written consent, participants were instructed to press “m” for target present and “v” for target absent. The trial begins by showing the memory set (see Figure 1), on a display. A fixation cross is shown for 1 s in the center of the screen. This is then followed by the search screen (see Figure 1). The visual set contained at most 1 memory set item. Subsequent trials are promptly initiated after a response, or, after 7 s if no response is given. Target and context were present in 50% of trials. Completion of the 112 trials automatically triggered the de-briefing slides. Trials were randomized and showed one of the 4 combinations of conditions—*either target present or target absent conditions in a context absent or context present trial*. The completion time was approximately 20 min.

#### 2.1.4 Design and data analysis

First, a descriptive analysis was conducted to assess the data’s statistical metrics to provide validation of the experiments’ properties and distributions delivered in an online medium. Second, a preliminary analysis was conducted to satisfy the data assumptions necessary for the main analyses. Pre-analysis checks examined outliers, homoscedasticity, normality of residual errors, and sphericity by inspecting Cook’s distance, the distribution of the residual error, and Mauchly’s test. Where Mauchly’s test indicated that the sphericity assumption had been violated (here, for variables with 2+ levels), Greenhouse–Geisser correction was applied. When significant and variance–covariance matrices were not homogenous, conservative Pillai’s trace was reported. No outliers (maximum Cook’s distance surpassing 1) were identified. For simple linear regressions, collinearity was assessed. No correlations above *r* > 0.9 were observed. By visually inspecting scatterplots of residuals against independent variables, no systemic pattern was observed (homoscedastic). In addition, examining residuals’ histograms, all conformed to an acceptable departure from normality.

To identify potential large differences being associated with one of the images used in a given condition, the following process was followed: First, a one-way ANOVA over images was conducted per condition {context present, MSS, VSS, target present} with formula rt. ~ stimulus. For example, for {context present = 1, MSS = 1, VSS = 4, target present = 1}, there were four stimuli answered by all participants, and the ANOVA is aimed to distinguish if there were one or some of the stimuli behaving differently than the others. Then, if the *F* value was larger than 20 with a corresponding *p*-value smaller than 0.05, *post-hoc* comparisons using Tukey’s HSD test (alpha = 0.05) were used to determine which of the stimuli would be discarded. For the stimulus with the highest mean difference compared to the others, only one stimulus was discarded per condition at most to keep data balancing. This resulted in 6 out of 112 images being discarded. For counterbalancing, the trial order was randomized across participants in each experimental task. Each participant experienced a unique sequence. The data were retrieved from https://pavlovia.org/ to Excel files and matched by participant ID.

To illustrate the relationships between reaction times and set size, linear and logarithmic regression models were constructed in Python with NumPy (version 1.23.5) for visual search and memory search. These models were used to fit each participant and condition ({MS, *VS*} × {CP, CA} × {linear, log}).

Linear mixed models (LMMs) were used on the combined data from all participants to compare models for both visual search (*VS*) and memory search (MS). For each search type, four models were constructed: two with a linear term for set size and two with a logarithmic term. Two of these models included context and set size (VSS for visual search, MSS for memory search) as fixed effects, their interaction, and participants as a random intercept [e.g., RT ~ VSS * Context + (1|Participant)]. We also specified a baseline model of decreased complexity that only included context as a fixed effect [*RT ~ Context + (1|Participant)*]. To assess the sequential decomposition of the contributions of fixed effect terms, we used likelihood ratio tests, which allowed us to compare models of different complexity (Burnham and Anderson, 2004; Bates et al., 2015). For model selection, we used the Akaike information criterion (AIC). This approach allowed for a direct comparison of linear and logarithmic models while accounting for the repeated measures design of the study (Bates et al., 2015).

Python (version 3.10.5) was used for general data management and analysis. SciPy (version 1.12.0) was used to compute Pearson correlation coefficient. NumPy’s polyfit was employed for linear regressions. Statsmodels (version 0.14.1) was used for the ANOVA table and *post-hoc* analyses. Although statsmodels includes mixed models, we opted for lme4 (version 1.1.35.1) in R (version 4.3.2) for linear mixed model analysis as it is widely used and has strong community support. To compare models of increasing complexity, we used the anova() function from the lme4 package (version 1.1.35.1). Scikit-learn (version 1.3.0) was used for its ParameterGrid implementation, which facilitated dividing data between conditions.

### 2.2 Results

#### 2.2.1 Online validation of experiments

Descriptive statistics of the hybrid search show properties consistent with previous lab-based experiments in literature, conferring adequate online validation. Consistent with Wolfe (2012a), this task saw a mean accuracy well above the chance level (*M* = 67%, SEM = 1%). The characteristic decline in accuracy when set sizes increase in divergent memory-mapping conditions was also observed: visual set size: VSS1 = 75% to VSS8 = 55%; and memory set size: MSS1 = 97% to MSS8 = 58%.

#### 2.2.2 Context effects and trial-by-trial memorization in hybrid search

In this section, the following abbreviations will be used for orderliness—target present (TP); target absent (TA); context present (CP); and context absent (CA). Across all conditions, targets were correctly detected on 67% (SEM:1%) of target present trials, and false alarms were produced on 26% (SEM:0.9%) target absent trials. The corresponding discriminability d-prime was 1.07.

For RT analyses, from a total of 12,320 trials across 110 participants, we excluded trials with no answer within the maximum time allowed (7 s) (*N* = 217). Very short responses (less than 200 ms) were only recorded for four trials and were not excluded from the data. Finally, we kept only correct trials which gave us a final sample of 8,042 trials (3,850 target present trials).

Linear mixed models with random intercepts per participant were used to control for repeated measurements. The inclusion of the variables of interest [VSS, MSS, log(VSS), log(MSS)] and their interaction with Context was evaluated by comparing these models [e.g., RT ~ VSS * Context + (1|Participant)] with the baseline model, containing only the Context as a fixed effect [*RT ~ Context + (1|Participant)*]. All variables of interest yielded significant improvements in log-likelihood over the baseline model [VSS: Δχ2 (2 df) = 215, *p* < 2.2e-16; log(VSS): Δχ2 (2 df) = 130, *p* < 2.2e-16; MSS: Δχ2 (2 df) = 320, *p* < 2.2e-16; log(MSS): Δχ2 (2 df) = 459, *p* < 2.2e-16]. In this context, the χ^2^ statistics represent the difference in deviance between successive models.

**Figure 2:**
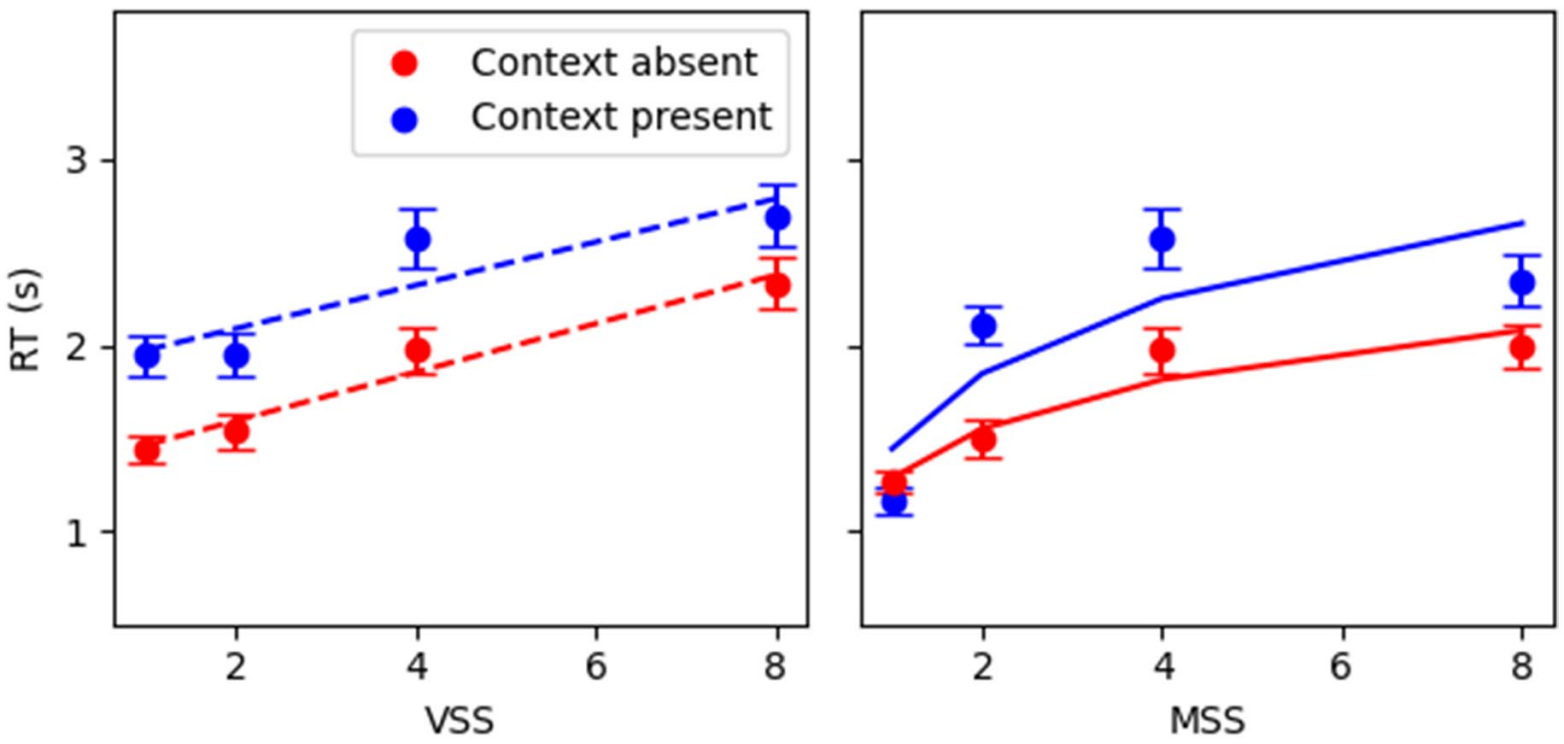
RT as a function of visual and memory set sizes for target present in correct-only trials. **(A)** Visual search target present; **(B)** memory search target present. In RT x VSS, MSS was fixed at SS4; in RT x MSS, VSS was fixed at SS4. Red squares denote context present (CP), and blue circles context absent (CA) conditions. Error bars denote 95% CI. Dashed/continuous lines depict linear/logarithmic fits, respectively. Equations for each condition: *VS*: *y* = (0.13 ± 0.01)*x + (1.35 ± 0.05) (CA); *y* = (0.13 ± 0.02) + *x + (1.84 ± 0.06) (CP); MS: *y* = (0.40 ± 0.03)*log(x) + (1.28 ± 0.04) (CA); *y* = (0.63 ± 0.04)*log(x) + (1.43 ± 0.05) (CP). The slope and the intercept were the mean ± SEM from the individual fits.

Linear and logarithmic dependencies were estimated for RT as a function of visual and memory set sizes (VSS and MSS, respectively) on a single-participant basis. For visual search, the classic linear dependence between RT and visual set size was replicated (Context Absent *R*^2^ = 0.54 ± 0.03; Context Present *R*^2^ = 0.49 ± 0.03). For memory search, a positive logarithmic relationship between RT and memory set size was observed (Context Absent *R*^2^ = 0.54 ± 0.03; Context Present *R*^2^ = 0.54 ± 0.03). Figure 2 exhibits the regression fits constructed for the average RT as a function of set sizes. The Akaike information criterion values were used to directly compare the linear and logarithmic models. Given the improvements in goodness of fit shown by models containing set size over the baseline model, comparisons were made for the full models. In visual search, the AIC value for the linear model (AIC_vs_linear: 10636) was lower than that for the logarithmic model (AIC_vs_log: 10717), indicating that the linear model in visual search better fits the data. Conversely, in memory search, the AIC value for the logarithmic model (AIC_ms_log: 10388) was lower than that for the linear model (AIC_ms_linear: 10531). This indicates that the logarithmic model in memory search provides a more parsimonious fit to the data than the linear model.

Correct-trial RT analysis (Figure 2) presented a positive slope with visual set size (*p* < 0.001, Table 1), larger RTs when the context was present (*p* < 0.001, Table 1), and no interaction between them (*p* = 0.13, Table 1) indicating that, for target present trials, both context conditions have similar efficiencies. Moreover, the main effects were still significant in a larger model including target absent trials (*p* < 0.001, Supplementary Table S2). In this case, the interaction showed a significant effect (*p* < 0.01, Supplementary Table S2) pushed by target absent trials (Supplementary Figure S3). The target presence showed no significant main effect but significant interactions with the other co-variables.

**Table 1:** Search efficiency.

		RT		
Predictors		Estimate	CI	*p*-value
Visual search	(Intercept)	1.23	1.11–1.34	<0.001
	Context present	0.49	0.37–0.62	<0.001
	VSS	0.12	0.10–0.14	<0.001
	Context present × VSS	−0.02	−0.05 − 0.01	0.133
	ICC (*N* = 110)	0.15		
	AIC	10,636		
Memory search	(Intercept)	1.27	1.16–1.38	<0.001
	Context present	0.10	–0.01 – 0.22	0.084
	log(MSS)	0.38	0.32–0.44	<0.001
	Context present × log(MSS)	0.24	0.15–0.33	<0.001
	ICC (*N* = 110)	0.16		
	AIC	10,388		

Both LMMs were built using the single trials from all participants with participants as random effects (N observations = 3,850). ICC, intraclass correlation coefficient. AIC, Akaike’s information criterion.

When considering memory search, the context did not present a significant effect by itself (Table 1) but an interaction with the logarithm of the set size log(MSS) (*p* < 0.0001, Table 1) indicating stronger log(MSS) effect when the context was present. The log(MSS) had a significant main effect by itself (*p* < 0.0001, Table 1). The significant effects were replicated when also considering the target absent trials (Supplementary Table S2), as well as the main effect of context and the main effect of target presence.

Similar models were built for accuracy as the dependent variable. There were significant main effects of visual set size and memory set size (Supplementary Table S3). No significant main effect of context, or interaction with visual/memory set sizes was observed. This suggests that participants followed a strategy in which they spent more time to locate each item but the extent to which they explored the image was the same. Both visual set size and memory set size odds ratios indicate a significant negative relation with the accuracy (odds < 1).

### 2.3 Discussion

Experiment 1 aimed to investigate the potential effects of context and trial-by-trial memorization on hybrid search performance. The results replicated the characteristic RT signatures seen in the existent lab-based literature (see Wolfe, 2020), as well as the typical decrease in accuracy as set sizes increased, consistent with results observed in previous hybrid search studies (Drew et al., 2017). Generally, as RT rose, accuracy diminished. Trial-by-trial memorization and context did not *qualitatively* affect RT. Whereas in visual search RT increased linearly as the visuals set size rose, in memory search RT increased logarithmically as memory set size escalated, with log fits exhibiting a smaller AIC score, thus providing a more parsimonious fit to the data, than linear fits. In target present conditions, the results *qualitatively* replicated the characteristic RT signatures of hybrid search even when context was present, and memorization occurred trial-by-trial.

In visual search, the canonical linear relationship between reaction time and set size seen in serial visual search literature and hybrid search literature was replicated (Treisman and Gelade, 1980; Wolfe, 2007, 2012a), whereas in memory search, the logarithmic relationship between reaction time and memory set size seen in traditional memory search studies and hybrid search was also observed here (Wolfe, 2012a; Nosofsky et al., 2014b; Boettcher and Wolfe, 2015). Memorization of all-new targets trial by trial does not seem to qualitatively affect the way attention is deployed and visual information is processed in non-efficient visual search (Shiffrin and Schneider, 1977).

Indeed, Schneider and Shiffrin (1977) demonstrated how manipulating memory lists to change (all-new mapping) or remain the same [consistent-mapping conditions—as seen in Wolfe (2012a) and existent hybrid search literature] on every trial provokes dramatic differences in accuracy patterns and RT efficiency in hybrid search but not RT *signatures*. A previously memorized target set used repeatedly throughout visual search trials can create memory reinstatement effects in memory search—that is, helps increase the distinction between memory representations and distractors (Nosofsky, 1986). Nosofsky et al. (2014b) found mean RTs are much faster, and error rates are much lower in the consistent-mapping condition than in an all-new condition in a memory search task. Consistently, this study observed lower accuracy in comparison with previous hybrid search studies, which had pre-tested fixed memory sets throughout trials (e.g., Wolfe, 2012a; Cunningham and Wolfe, 2014).

When context was present, we observed higher RT intercepts and similar RT slopes compared to context absent conditions. The context in our stimuli did not violate scene syntax or scene semantics. Prior studies have shown that syntactic and semantic violations affect attention allocation in scene processing. Indeed, syntactic inconsistencies (e.g., floating objects) and scene inconsistencies (e.g., searching for a toaster on the floor) have been shown to impair scene guidance of attention when compared to semantically consistent objects in scenes, such as a toaster on a table (Wolfe, 2020). Differences in search slopes might emerge if distractors in our stimuli set were placed in more implausible locations than the targets, as observed by Neider and Zelinsky (2006) in a visual search task. Therefore, future studies using stimuli sets with varying degrees of semantic association between the context and memory set items are needed to uncover the influence of context on hybrid search.

While the main analyses were conducted on target present trials, we also analyzed target absent trials. In target and context absent conditions, RT shapes replicated the linear dependence between visual set size and RT and the logarithmic dependence between memory set size and RT (Wolfe, 2012b; Boettcher and Wolfe, 2015). Accuracy overall also decreased as set sizes increased. However, the quality of the model adjustments in these conditions was worse than the ones when the target was present. As explored below, this could be linked to participants’ adoption of adaptive strategies for search termination.

## 3 A drift-diffusion computational model of hybrid search

### 3.1 Methods

**Figure 3:**
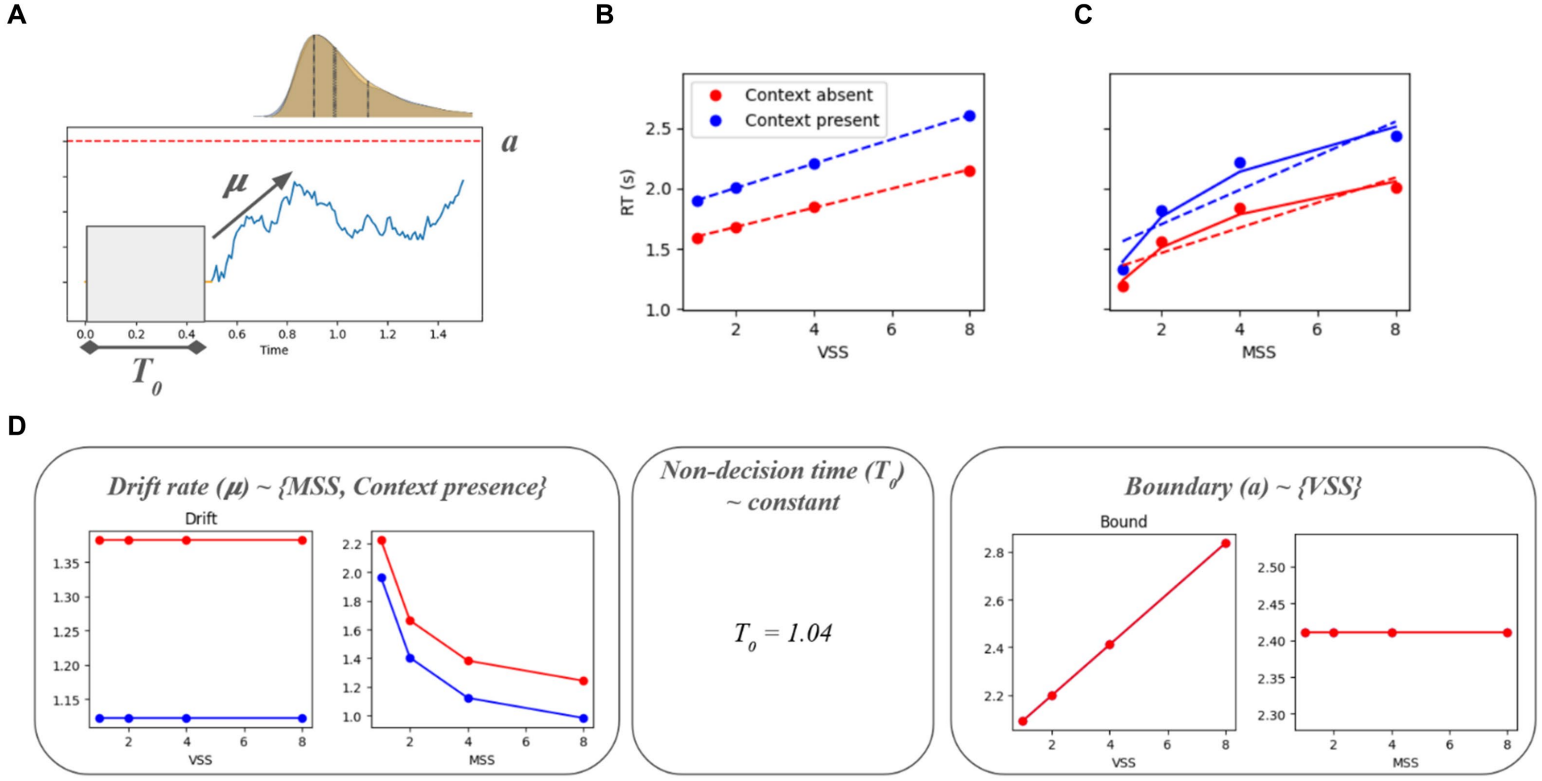
Interaction between the parameters of a drift-diffusion model (DDM) and the visual search/memory search parameters: The drift interacts with the memory set size and the context, while the decision boundary interacts with the visual set size. The DDM was fit to the target present trials. The RT simulations (averaged across 10,000 per condition) show similar results as what was seen in Figure 2.

A DDM was implemented using the Python package pyddm 0.7.0 (Shinn et al., 2020). At first, the model was fitted using all the target present data using pyddm.models.loss.LossRobustLikelihood as the loss function and differential evolution as the fitting method (the rest of the parameters were the default). In this case, the goal was not to find the best, generalizable model but to explain its parameter values in terms of the experimental variables (MSS, VSS, and context). This would allow a better interpretation of the effects in terms of the model’s parameters. The parameters considered were the drift rate (μ), the boundary (a), and the non-decision time (t0). To keep the model simple and maintain interpretability, the drift rate was assumed to depend only on the MSS and the context, while the boundary was assumed to depend on the VSS (Figure 3). There were no constraints on the non-decision time. As a form of validation, RT simulations were performed to assess whether the RT × VSS and RT × MSS curves were like the experimental data or not. In the second step, the model was fitted including target absent data, and the boundary and non-decision time could vary with the target’s presence (Supplementary Figure S4).

### 3.2 Results

Figure 3 shows the results of a drift-diffusion model fit for each of the conditions. Panel A describes mu (drift rate), a (boundary), T0 (non-decision time), and how they affect a typical drift-diffusion model. A comparison of Panels B and C with Figure 2 shows that the main behavioral effects can be replicated, although an additional significant context X VSS interaction emerges. Panel D shows how those parameters relate to each experimental variable (MSS, VSS, and context). The drift rate has a multiplicative inverse relationship with the memory set size, while the context applies an intercept to the drift rate. The non-decision time remains constant across experimental conditions. The boundary is linear in terms of the visual set size. In this model, there is a constant relationship between boundary and memory set size, boundary and context, and drift rate and visual set size.

### 3.3 Discussion

Previous computational models of memory/visual search (Moran et al., 2013; Nosofsky et al., 2014b) have provided useful information related to both search processes and termination, which was later incorporated into general models of visual search, such as Wolfe’s Guided Search 6.0. However, these models are particularly suitable for setups where the stimuli are artificial (i.e., white background with geometric figures) and are not defined in setups with natural scenes (where there is an inherent correlation between different regions in the image as well as added noise). This can be overcome by using data-driven approaches such as the drift-diffusion model we implemented here.

In a drift-diffusion model, the drift rate is associated with the amount of information a subject accumulates in a single time step. A higher drift rate would result in a lower RT. We observed that the drift rate goes down as the MSS goes up in a logarithmic fashion (Figure 3). One simple interpretation is that the higher the MSS, the more time is needed to process/remember the memory set, so in a fixed time interval participants will be less certain of what they are looking for when the MSS is large. The context applies a bias to the drift rate. This could be related to the fact that when looking at objects with a white background, there will typically be a few fixations in the background. However, when there is context present, there are contextual cues in the background that guide our search and the stimulus is noisier, which would add an overhead to the processing of the image.

The boundary is related to the amount of evidence needed to make a decision. The further it is from 0, the higher will be the RT. We observed that the boundary is linearly proportional to the visual set size (Figure 3). Interpreting the evidence as the proportion of the image explored until the target object is found, more objects will require increased attention when the visual set size is high, which in turn would result in a larger portion of the image being explored. This means that the higher the set size, the more evidence is needed to decide that the object has been found.

## 4 Experiment 2: a change detection and a go/no-go task to explore individual differences in hybrid search

### 4.1 Methods

#### 4.1.1 Participants

The participants were the same as in Experiment 1. Typically, Experiment 2 was conducted after a short break following Experiment 1.

#### 4.1.2 Materials

Visual working memory capacity was measured using the classic change detection task (CDT) adapted from previous studies (Xu et al., 2018; Balaban et al., 2019). It involved 120 trials with set size 4 (*N* = 60) and set size 6 (*N* = 60) of colored blocks. To measure inhibitory control, the well-established go/no-go (GNG) task was employed. It was based on the parameters of previous studies (Wessel, 2018; Young et al., 2018). To isolate inhibitory control from other cognitive processes, the 150 trials varied between a blue circle in go trials (*N* = 120) and an orange circle in no-go trials (*N* = 30).

#### 4.1.3 Procedure

Trials began with four or six color blocks, followed by a fixation cross and, subsequently, a detection screen with one color block (for timeframes, see the Supplementary Figure S1). If the same color and location as in the initial display appeared, the “k” key was pressed. If a different color and/or location popped up, “l” was pushed. The next trial automatically started after a response without feedback. Changes occurred in 50% of trials. Once the 120 CDT trials were completed (approximately 10 min), the Go/No-Go task instructions appeared. After four practice trials with feedback, the Go/No-Go task started by displaying either blue (go stimuli; size [0.15, 0.15]cm) or orange circles (no-go stimuli; size [0.15, 0.15]cm) at a 4:1 go/no-go ratio. For go stimuli, the space bar was pressed, and for no-go stimuli, that action had to be inhibited. With an intertrial interval of 450 ms, 150 trials lasted approximately 3 min (see the Supplementary Figure S2). Part 2 lasted approximately 15 min.

#### 4.1.4 Data analysis

Simple linear regressions were performed to assess the potential effects of individual differences in visual working memory capacity and inhibitory control on hybrid search performance. Here, the independent variables were the memory capacity K and the response bias c (see Supplementary material), and the dependent variables included the RT slopes and intercepts for each condition (context absent or present, and target absent or present). Age was also checked for its potential impact on search; however, it did not show a significant result. While some measures in this study are from direct observations, visual working memory capacity and inhibitory control are measured by K and c, respectively. K is the estimate of an individual’s VWM capacity, whereas c is seen as a measure of decision/response bias. These are calculated through Cowan’s (2001) formula and SDT analysis (Green and Swets, 1966), respectively.

Scikit-learn (version 1.3.0) and statsmodels (version 0.14.1) in Python (version 3.10.5) were used for data analysis.

### 4.2 Results

To ensure the validity of the visual working memory capacity K on the change detection task experiment, the normality of K was assessed using a Kolmogorov–Smirnov normality test. As in previous studies (Balaban et al., 2019), K was found to be normally distributed [D (110) = 0.065, *p* > 0.05]. This study’s K replicates two pivotal characteristics concerning VWM capacity—(1) capacity-limited characteristic (*M* = 2.31) and (2) significant individual differences denoted by *SD* = 0.82 (Balaban et al., 2019). It is consistent with China-based (K:2.14) (Xu et al., 2018) and American studies (K:2.55) (Fukuda et al., 2016).

Signal detection theory analysis was applied to calculate the decision bias measure in the Go/No-Go task. A negative c mean (*M* = −0.23, SD = 0.58) was achieved, consistent with existing literature (Young et al., 2018).

A correlation analysis was performed to investigate the impact of individual differences in memory capacity K, decision bias c, and false alarms on hybrid search performance, measured through RT intercepts and slopes. For target present trials, none of the correlations (see Figure S5) were statistically significant, with values ranging from −0.18 to 0.18. However, for target absent without context, memory capacity and RT slopes were positively correlated (*R* = 0.18, *p* < 0.05). Participants’ decision bias c did not predict RT in any condition when the target was present, but it did predict RT intercepts when the target was absent (*R* = 0.22 for context present and *R* = 0.20 for context absent). Nevertheless, all these correlations explained little variability in hybrid search performance (maximum *R*^2^ = 0.05).

### 4.3 Discussion

Experiment 2 assessed the possible interplay of visual working memory capacity and inhibitory control in hybrid search.

The general lack of relationship between visual working memory capacity (VWM) and hybrid search performance measures is inconsistent with accounts that propose that search/target templates reside in VWM to (1) bias attentional deployment to goal-relevant objects (Desimone and Duncan, 1995; Soto et al., 2008) and (2) be compared to suitor objects (Bundesen, 1990). If that was so, higher VWM capacity individuals, supposedly having larger storing capacities or better ability in manipulating attentional resources (see Luck and Vogel, 2013), would search more efficiently as set sizes increase, than observers with lower VWM capacity (Sobel et al., 2007). However, these results are partially consistent with the sparse hybrid search literature that previously assessed VWM capacity’s role. Indeed, Drew et al. (2016) used a dual-task paradigm with CDTs in between HS tasks and found that performing a hybrid search task diminished VWM capacity by a fixed amount (e.g., one slot) regardless of set size variation. Hence, if it is assumed that a “one-item” channel/path must successively move visual items to long-term memory, VWM interaction would not be dependent on set size, that is, only a fixed amount of each individual’s VWM capacity would be used for the task. This would be consistent with the largely non-significant results seen here between VWM capacity differences and hybrid search RT *slopes*.

An important difference between this study and other studies including (Drew et al., 2016) is that memory sets here were not kept constant, changing trial by trial. In the visual search literature, some dual-task studies indicated that loading VWM, with a change detection task embedded in a *VS* task, solely impacted search when target sets changed per trial, not when they were kept constant (Woodman et al., 2007; Woodman and Arita, 2011). Behaviorally, they observed a slowing of RT slopes when loading working memory. However, electrophysiological data suggested an effect on working memory representation maintenance (Fukuda and Vogel, 2009). While the results presented are inconsistent with these findings, this points to the importance of evaluating electrophysiological data alongside behavior to pull apart the meaning of behavioral observations.

Studies investigating the effect of individual differences in inhibitory control (IC) on search, especially those with direct inhibitory control measures (Clarke et al., 2022), are markedly scarce. Given inhibition’s clear importance in visual search (Beck and Kastner, 2009) and search termination (Moran et al., 2013), this study investigated the IC’s role in hybrid search. The results showed that individuals with higher IC (lower negative bias) had significantly higher RT intercepts than participants with lower IC only when the target was absent (Supplementary Figure S5). These results suggest that more inhibitory control is needed to remain in search when there is no target on the visual display. This is consistent with Moran et al. (2013) competitive-guided search model that advances inhibitory links between the priority search map and the quitting unit that terminates the search when selected, and other memory search models that propose a diffusion stage with laterally inhibiting racing target items (i.e., Cunningham and Wolfe, 2014; Nosofsky et al., 2014b). In target present trials, the activation of the priority map is automatically increased, which leads to higher inhibition of the quit unit in comparison to target absent trials (Moran et al., 2013). In other words, target absent conditions inherently increase quitting probability.

## 5 General discussion and conclusion

This study contributes to the understanding of various elements involved in typical real-world search scenarios, where multiple targets are searched for, contextual information is available, and searching occurs after a single exposure. Unlike traditional search scenarios with items presented against a blank background, the fundamental reaction time signatures of hybrid search—linear increase with visual set size and logarithmic increase with memory set size—are preserved. Individual differences in working memory and inhibitory control only showed a modest impact on search termination when the target was absent, aligning with established visual search models such as the guided search model. In addition, we showed that a simple computational drift-diffusion model can reproduce the primary behavioral results of a hybrid search. To our knowledge, our study is the first hybrid search study to present search items against a photorealistic scene background rather than the commonly used blank backgrounds. This approach introduces a more realistic context, which is crucial for understanding search behaviors in real-world settings. Indeed, our findings have potential implications for real-world tasks, such as airport security screening, where officers must remember a set of prohibited items (memorized targets) among numerous distractors, and medical imaging analysis, where radiologists use their expertise to identify abnormal patterns in images.

While this study has made contributions toward a better understanding of search in real life, it also had some limitations. First, this study did not systematically manipulate the categories in memory sets (i.e., in each trial, targets and distractors belonged to the same category) and scene semantics/syntactics (i.e., there were no objects placed in scenes with semantic/syntactic violations). Second, the experiment design does not allow direct comparisons between the efficiency and accuracy in all-new mapping and consistent-mapping conditions as this would require a separate comparison group. Since the mapping of the stimuli was not manipulated, the comparisons made here are related to the qualitative nature of the relationship between reaction times and set sizes. Third, the amount of time observers fixated on *individual* items was not controlled. This can also affect memory strength and working memory consolidation for each target item (Donkin and Nosofsky, 2012). Future studies could include eye tracking to better understand how memory strength impacts memory search via dwell times, and how observers encode target items in memory. This can help disentangle VWM’s role in hybrid search and understand memory strength impact, which is not controlled for in the existent hybrid search corpus (Nosofsky et al., 2014b). This could also give more information to the model to work with, or even allow the use of scanpath prediction models in conjunction with, a drift-diffusion model (Travi et al., 2022). Fourth, the current drift-diffusion model does not explain the variability across subjects or stimuli. Finally, as an online study, environmental factors were uncontrolled, and attentional engagement throughout the experiment could not be assessed. This could have impacted the search in several ways including participants’ appraised value of continuing the search if there were external attentional demands present. Contextual information in search should be further investigated in lab-based studies to strengthen the results reported here.

Altogether, our study exposes an intricate interplay of various behavioral mechanisms in hybrid search. While the results reported here provide some valuable insights, some key questions remain unanswered, particularly concerning the influence of scene semantics on hybrid search and the little role that visual working memory seems to have on hybrid search. To address these intriguing aspects, future research can leverage recent technical advancements, such as concurrent M-EEG and eye movement recordings (Dimigen and Ehinger, 2021; Care et al., 2023). The integration of these cutting-edge methodologies promises to unveil the underlying physiological mechanisms driving hybrid search, further deepening our understanding of this complex cognitive phenomenon.

## Data Availability

The datasets and code presented in this study are available on https://github.com/NeuroLIAA/HybridSearch_Online/. Experiments can be accessed at https://run.pavlovia.org/mison/cdt_gng_ius12/html/ and at https://run.pavlovia.org/isonlab/hybrid_search/html/.
