# Supplementary material for: Investigating the effects of context, visual working memory, and inhibitory control in hybrid visual search
Source: Front Hum Neurosci. 2024 Aug 27;18:1436564. doi: 10.3389/fnhum.2024.1436564 (PMC11384996; doi:10.3389/fnhum.2024.1436564)
Supplement: Supplementary file 1 [file Data_Sheet_1.PDF]

Supplementary Material

1 Supplementary Data

Supplementary Material should be uploaded separately on submission. Please include any supplementary data, figures and/or tables.

Supplementary material is ngot typeset so please ensure that all information is clearly presented, the appropriate caption is included in the file and not in the manuscript, and that the style conforms to the rest of the article.

2 Supplementary Figures and Tables

For more information on Supplementary Material and for details on the different file types accepted, please see here.

2.1 Supplementary Figures

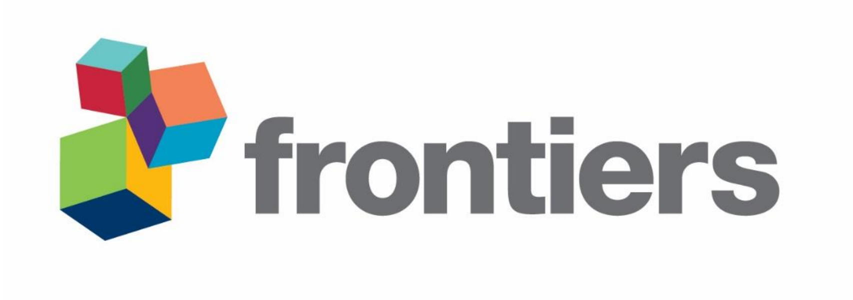

Supplementary Figure 1. The figure legends are required to have the same font as the main text, 12 point normal Times New Roman, single spaced. Please use a single paragraph for each legend and prepare the figures keeping in mind the PDF layout.

Change Detection Task (CDT) and Go-No Go (GNG) Procedure and Formulas

Figure S1.

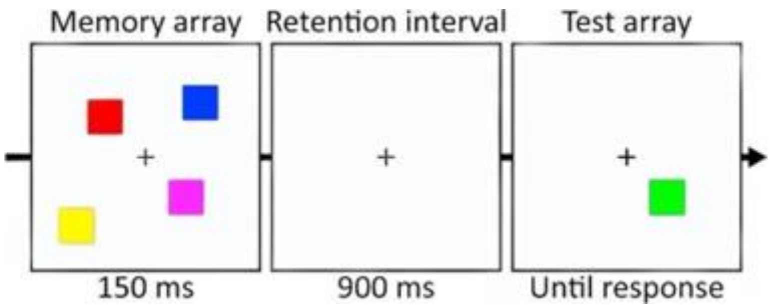

CDT model trial of set size 4 with a change in the test array.

$$K = N \times (HR - FA)$$

$$HR = \frac{\text{\#correct responses in change trials}}{\text{\#change trials}}$$

$$FA = \frac{\text{\#incorrect responses in no-change trials}}{\text{\#no-change trials}}$$

Where N denotes the number of trials, HR is the hit rate and FA the false alarms rate.

Figure S2.

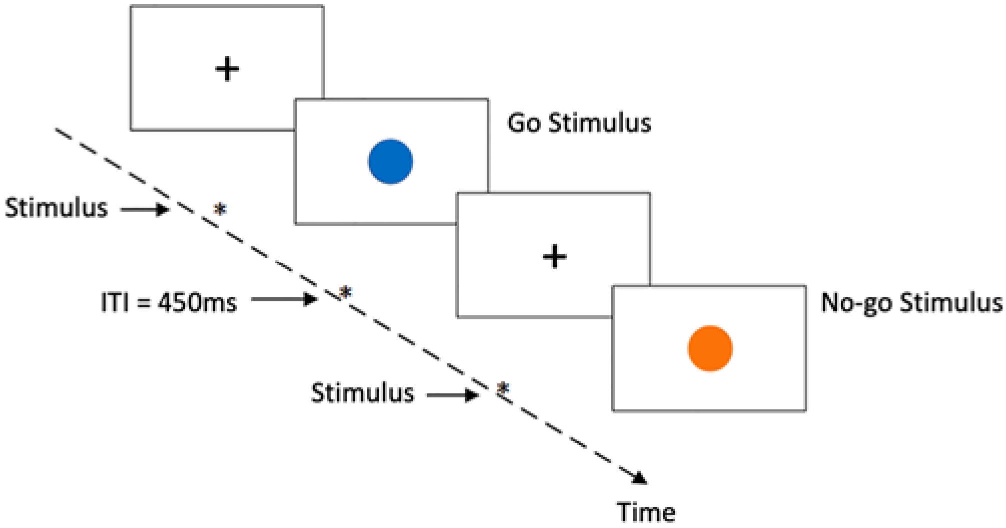

GNGT example of trials throughout time.

$$c = -\frac{1}{2}\left[z(HR) + z(FA)\right]$$

$$HR = \frac{\text{\#go response in go trials}}{\text{\#go trials}}$$

$$FA = \frac{\text{\# go response in no-go trials}}{\text{\# no-go trials}}$$

Here, c is the response bias. Z(HR) the z-transformed hit rate and Z(FA) the z-transformed false alarm rate. When FA are 0 or 1, then we adopted the corrections suggested by Stanislaw & Todorov, 1999 (substituting 0 for 0.5/N, and 1 for 1-0.5/N, where N is the number of target absent trials).

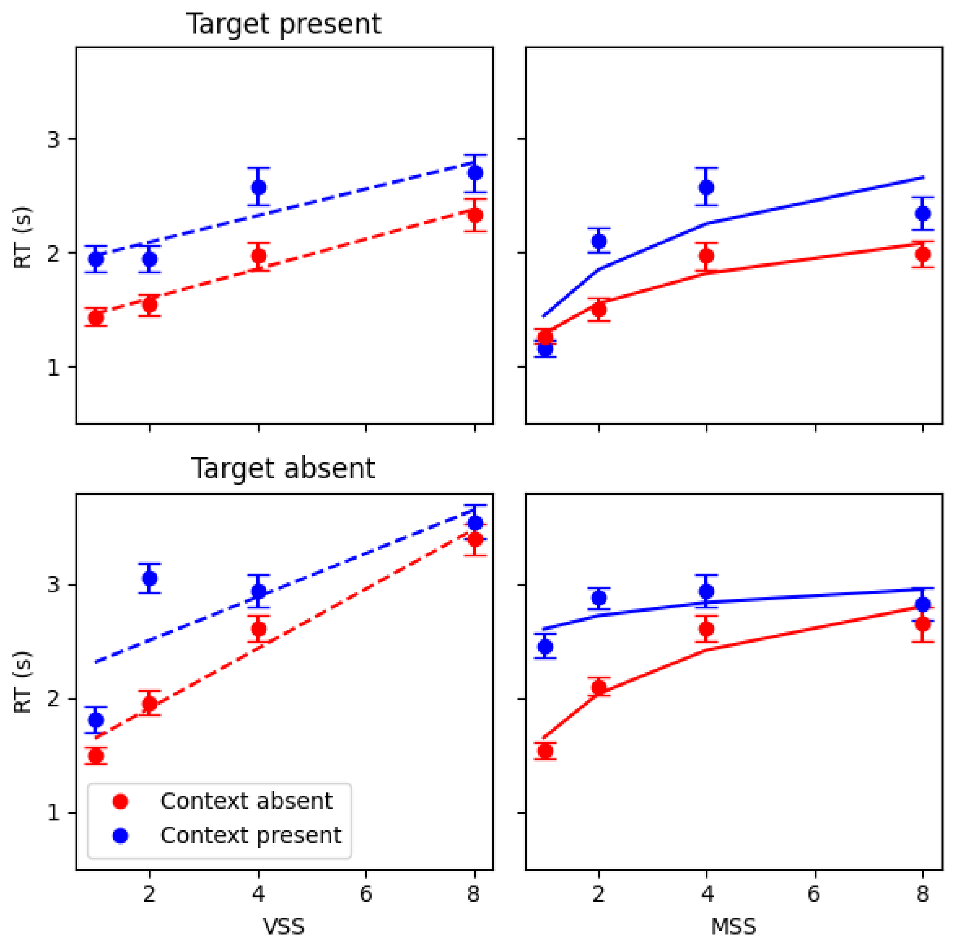

Figure S3. Context Effects in Hybrid Search *RT as a function of visual and memory set sizes for target present (TP) in panels A and B, target absent (TA) in panels C and D, in correct trials. Red squares denote Context Present (CP), and cyan circles context absent (CA) conditions. Error bars denote 95% CI. Dashed lines depict linear fits, while continuous lines portray logarithmic fits. Panels differentiate conditions: A) Visual Search Target Present; B) Memory Search Target Present; C) Visual Search Target Present; D) Memory Search Target Absent. Equations for each condition – VS: y = 0.13*x + 1.33 (TP/CA); y = 0.12*x + 1.86 (TP/CP); y = 0.26*x + 1.38 (TA/CA); y = 0.19*x + 2.12 (TA/CP). MS: y = 0.38*log(x) + 1.29 (TP/CA); y = 0.58*log(x) + 1.44 (TP/CP); y = 0.55*log(x) + 1.65 (TA/CA); y = 0.17*log(x) + 2.60 (TA/CP). In RT x VSS, MSS was fixed at SS4; in RT x MSS, VSS was fixed at SS4.*

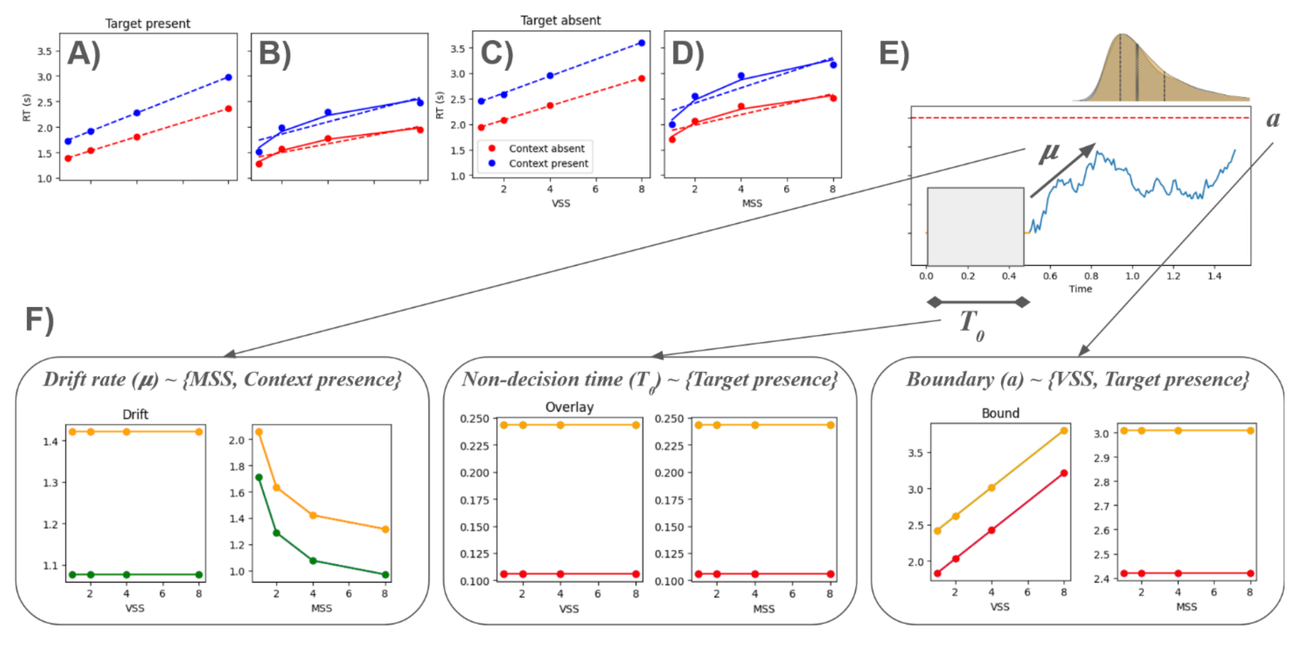

Figure S4. *Interaction between the parameters of a DDM model and the visual search/memory search parameters: the drift interacts with the MSS and the context flag; the decision boundary interacts with the VSS and the target flag; the non-decision time interacts with the target flag. The DDM was fit to the correct trials. The RTs simulations (averaged across 10000 per condition) show similar results as what was seen in Supplementary Figure 2.*

Table S1. *Goodness of fit using R2 for correct trials. The fit was done for each subject and then the R2 was averaged across subjects. The p-value was obtained using a wilcoxon test.*

		linear		Log-linear		
		R2	R2 std error	R2	R2 std error	p-value
Visual Search	TP/CP	0.49	0.03	0.46	0.03	0.13
	TP/CA	0.54	0.03	0.51	0.03	0.09
	TA/CP	0.50	0.03	0.59	0.03	0.00
	TA/CA	0.75	0.02	0.77	0.02	0.12
	AIC		21983.82		22178.32	
Memory Search	TP/CP	0.43	0.03	0.54	0.03	0.00
	TP/CA	0.51	0.03	0.54	0.03	0.01
	TA/CP	0.42	0.03	0.43	0.03	0.99
	TA/CA	0.53	0.03	0.59	0.0	0.00
	AIC		22578.46		22393.38	

Table S2. *Search efficiency. Both LMMs were built using the single-trials from all the participants (N observations = 8042).*

		rt		
Predictors		Estimate	CI	p-value
Visual Search	(Intercept)	1.20	1.09-1.32	<0.001
	Context present	0.88	0.76-1.00	<0.001
	VSS	0.25	0.23-0.27	<0.001
	Target present	0.02	-0.10-0.14	0.708
	Context present x VSS	-0.07	-0.10-(-0.04)	<0.01
	Context present x target present	-0.39	-0.57-(-0.22)	<0.01
	Target present x VSS	-0.13	-0.16-(-0.10)	<0.01
	Context present x target present x VSS	0.05	0.01-0.09	0.026
	ICC (*N = 110)*	0.17		
	AIC	20983.82		
Memory Search	(Intercept)	1.61	1.50-1.73	<0.001
	Context present	0.98	0.87-1.10	<0.001
	Log MSS	0.52	0.45-0.58	<0.001
	Target present	-0.34	-0.46-(-0.23)	<0.001
	Context present x log MSS	-0.36	-0.45-(-0.27)	<0.001
	Context present x target present	-0.88	-1.05-(-0.72)	<0.001
	Target present x log MSS	-0.15	-0.24-(-0.06)	0.02
	Context present x target present x log MSS	0.60	0.47-0.73	<0.01
	ICC (*N = 110)*	0.17		
	AIC	22393.38		

Table S3. *Search accuracy. Both GLMMs were built using the single-trials from all participants (N observations = 3850). ICC: Intraclass Correlation Coefficient. AIC: Akaike Information Criterion.*

		Correct response		
Predictors		Odds Ratios	CI	p-value
Visual Search	(Intercept)	3.23	2.67-3.91	<0.001
	Context present	0.96	0.76-1.23	0.771
	VSS	0.91	0.87-0.94	<0.001
	Context present x VSS	0.99	0.94-1.04	0.691
	ICC (*N = 110)*	0.06		
	AIC	7483		
Memory Search	(Intercept)	4.44	3.65-5.40	<0.001
	Context present	0.96	0.75-1.22	0.715
	MSS	0.84	0.81-0.87	<0.001
	Context present x MSS	0.99	0.94-1.05	0.738
	ICC (*N = 110)*	0.06		
	AIC	7366		

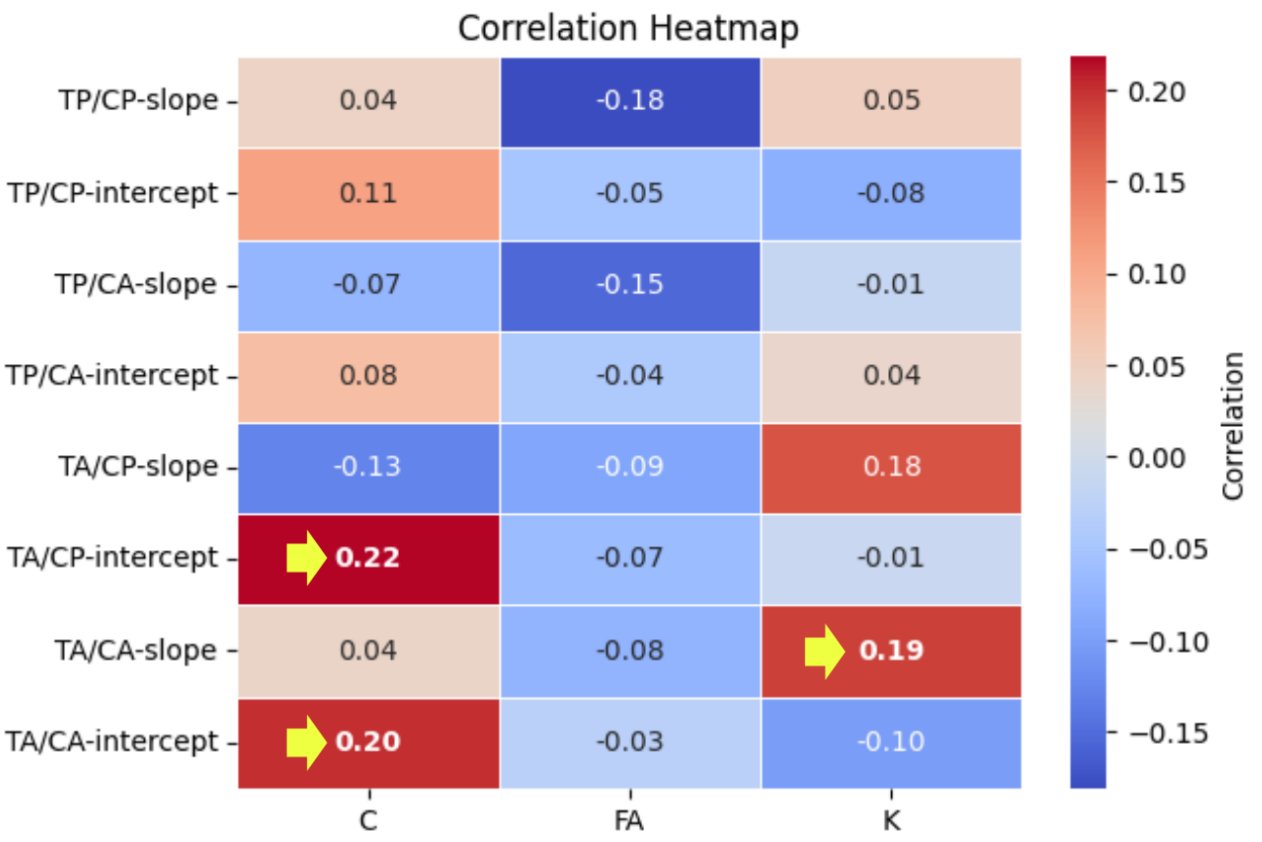

Figure S5. *Individual differences: each cell is the Pearson Correlation between a parameter (K,C,FA) and a fitness parameter (slope, intercept) for a given condition (TP/CP, TP/CA, TA/CP, TA/CA).*: uncorrected p-value < 0.05.*
